# Supercontinuum generation in scintillator crystals

**DOI:** 10.1038/s41598-024-84178-6

**Published:** 2025-01-04

**Authors:** Vaida Marčiulionytė, Gintaras Tamošauskas, Matas Šutovas, Audrius Dubietis

**Affiliations:** Laser Research Center, Vilnius University, Saulėtekio Avenue 10, LT-10223 Vilnius, Lithuania

**Keywords:** Supercontinuum generation, Infrared, High repetition rate, Scintillator crystals, Luminescence, Materials science, Optics and photonics

## Abstract

We present a comparative experimental study of supercontinuum generation in undoped scintillator crystals: bismuth germanate (BGO), yttrium orthosilicate (YSO), lutetium oxyorthosilicate (LSO), lutetium yttrium oxyorthosilicate (LYSO) and gadolinium gallium garnet (GGG), pumped by 180 fs fundamental harmonic pulses of an amplified Yb:KGW laser. In addition to these materials, experiments in yttrium aluminium garnet (YAG), potassium gadolinium tungstate (KGW) and lithium tantalate (LT) were performed under identical experimental settings (focusing geometry and sample thickness), which served for straightforward comparison of supercontinuum generation performances. The threshold and optimal (that produces optimized red-shifted spectral extent) pump pulse energies for supercontinuum generation were evaluated from detailed measurements of spectral broadening dynamics. The measured cut-off wavelengths at the short-wavelength side are in line with the general trend of blue-shifted spectral broadening on the bandgap of nonlinear material. All the nonlinear materials produced reasonable red-shifted spectral broadenings under conditions of optimal pump pulse energies, with the largest red-shift exceeding 2000 nm measured in GGG crystal. Our results revealed that GGG and BGO (which also had the lowest supercontinuum generation threshold) offer durable, optical damage-free performance at a laser repetition rate of 200 kHz, suggesting that these materials are good alternatives to YAG and KGW for low threshold, high average power supercontinuum generation in the near- and short-wave infrared spectral ranges. We also demonstrated that scintillating properties of bulk materials could be readily studied in the filamentation regime, via multiphoton excitation using near-infrared femtosecond laser pulses.

## Introduction

Since its discovery in 1970^1,2^, the state of the art of supercontinuum (SC) generation in bulk solid-state materials has reached a high level of maturity thanks to tremendous progress of ultrafast laser sources and fundamental understanding of the underlying physical processes^3,4^. The complexity of physical effects underlying SC generation is unveiled in the framework of femtosecond filamentation, which is a universal phenomenon arising from nonlinear propagation of femtosecond laser pulses in transparent materials^5^. In more practical terms, the attainable spectral extent of SC radiation depends on the material properties, such as linear and nonlinear indexes of refraction and energy bandgap, which together with the laser wavelength define the critical power for self-focusing (which has to be exceeded for a light filament to form) and clamping intensity of a light filament (which is proportional to the order of multiphoton absorption), respectively. The set of these basic parameters nicely explains the experimental data for a wide range of materials^6,7^, see also^8^, and references therein for the most recent results, demonstrating that the blue-shift of the SC spectrum scales with $U_g/\hbar \omega _0$, where $U_g$ stands for the energy bandgap of the material and $\hbar \omega _0$ denotes the incident photon energy. By contrast, the attainable red-shift of the SC spectrum is not so strictly defined and strongly depends on the focusing condition (the numerical aperture) of the pump beam even in the same nonlinear material. The experiments backed up with the numerical simulations showed that loose focusing of the pump beam favors spectral broadening toward the long-wavelength side, thus allowing optimization of the red-shifted content of the SC spectrum^9,10^.

Bulk-generated SC generation serves as an important asset to device applications, such as development of ultrafast optical parametric amplifiers^11^ and optical parametric chirped pulse amplifiers^12^, and finds a wide range of applications in diverse areas of ultrafast science, including pulse post-compression^13^, see also^14^ for a review, wave-form synthesis^15^, time-resolved spectroscopy^16^, coherent anti-Stokes Raman spectroscopy (CARS)^17–19^, and nonlinear microscopy^20^, to mention a few. The diversity of applications calls for optimization of relevant performance characteristics of bulk-generated SC, such as wavelength stability and timing jitter^21^ and carrier envelope phase noise^22^. On the other hand, a significant effort was dedicated for the search of new nonlinear and efficient materials for SC generation with near-IR pumping, in particular aiming at production of high spectral densities within desired wavelength ranges^23–31^, as well as for seeking robust operation of well-established and newly introduced nonlinear materials at very high laser pulse repetition rates, which are available with current state-of-the art femtosecond Yb-doped laser sources^28,30,32–36^. To this end, the most recent results demonstrate that several previously poorly explored materials possessing relatively narrow energy bandgaps, such as diamond, calcium tungstate ($\hbox {CaWO}_4$), potassium gadolinium tungstate (KGd($\hbox {WO}_4$)$_2$, KGW) and yttrium vanadate ($\hbox {YVO}_4$) produce SC spectra with remarkable red shifts despite very moderate spectral broadenings toward the short wavelength side, and exhibit robust, optical damage-free performances at MHz^28,30^ and even multi-MHz pulse repetition rates^35^.

In this Paper, we present a thorough comparative experimental study of SC generation with 180 fs, 1030 nm pulses from an amplified Yb:KGW laser in bismuth germanate ($$\hbox {Bi}_4$$$$\hbox {Ge}_3$$$$\hbox {O}_{12}$$, BGO), gadolinium gallium garnet ($$\hbox {Gd}_3$$$$\hbox {Ga}_5$$$$\hbox {O}_{12}$$, GGG), yttrium orthosilicate ($$\hbox {Y}_2$$$$\hbox {SiO}_5$$, YSO), lutetium oxyorthosilicate ($$\hbox {Lu}_2$$$$\hbox {SiO}_5$$, LSO), lutetium yttrium oxyorthosilicate ($$\hbox {LuYSiO}_4$$, LYSO) and lithium tantalate ($$\hbox {LiTaO}_3$$, LT) single crystals, whose performances were compared to yttrium aluminium garnet ($$\hbox {Y}_3$$$$\hbox {Al}_5$$$$\hbox {O}_{12}$$, YAG) and potassium gadolinium tungstate (KGd($$\hbox {WO}_4$$)$$_2$$, KGW). Although BGO, GGG, YSO, LSO and LYSO occasionally serve as laser hosts^37^, they are primarily identified as efficient scintillators, whose scintillation properties are studied quite well^38^. However, the nonlinear optical properties of these crystals and so their potential to serve as nonlinear materials for SC generation in particular, and any other experiments concerning nonlinear propagation of intense femtosecond laser pulses, remain very poorly studied. To date, GGG, LT^23,25^ and YSO^24^ were only sketchily tested for SC generation with Ti:sapphire laser pumping, however, to the best of our knowledge, none of the investigated materials were examined with Yb-laser pumping, especially for what concerns their performance at very high (tens to hundreds of kHz) pulse repetition rates.

## Materials and methods

All crystal samples were provided by Eksma Optics. The samples were undoped and uncoated and had equal dimensions of 5 $\times$ 5 $\times$ 10 $\hbox {mm}^{3}$, so their identical lengths enabled direct comparison of the SC generation performances. The relevant optical parameters: energy bandgaps, linear and nonlinear indexes of refraction and estimated critical powers for self-focusing of the investigated materials are listed in Table 1. Since no experimentally measured $\hbox {n}_2$ values of LSO and BGO were available, these values were calculated using two parabolic band model^39^. No published data of LYSO parameters was found in the literature, therefore these were taken equivalent to LSO, as suggested by comparative measurements of optical absorption, radioluminescence, etc., which were reported to be very similar in both materials^40^.

**Table 1:** Relevant optical properties of tested materials: $U_{g}$ is the energy bandgap, $n_{0}$ is the linear index of refraction at 1030 nm, as calculated from Sellmeier equations, $n_{2}$ is the nonlinear index of refraction, and $P_{cr}$ is the critical power for self-focusing.

Material	$U_{g}$ (eV)	$n_{0}$	$n_{2}$ ($10^{-16}$ $\hbox {cm}^{2}$/W)	$P_{cr}$ (MW)
YAG	6.5^41^	1.82^42^	6.13^43^	1.42
LSO	6.4^44^	1.78^45^	4.0$$^{*}$$, 7.6$$^{**}$$	2.24$$^{*}$$, 1.18$$^{**}$$
LYSO	6.4^40^	1.78^40^	4.0$$^{*}$$, 6.6$$^{**}$$	2.24$$^{*}$$, 1.36$$^{**}$$
YSO	6.14^46^	1.78^47^	6.1^48^	1.47
GGG	5.66^49^	1.96^50^	12.4^42^	0.65
LT	4.43^23^	2.14^51^	17^52^	0.43
KGW	4.25^53^	2.01^53^	16^54^	0.49
BGO	4.16^55^	2.06^56^	21$^{*}$	0.37

*Calculated using two parabolic band model^39^ **Estimated from the experimentally measured threshold energies for SC generation, this work.

**Fig. 1:**
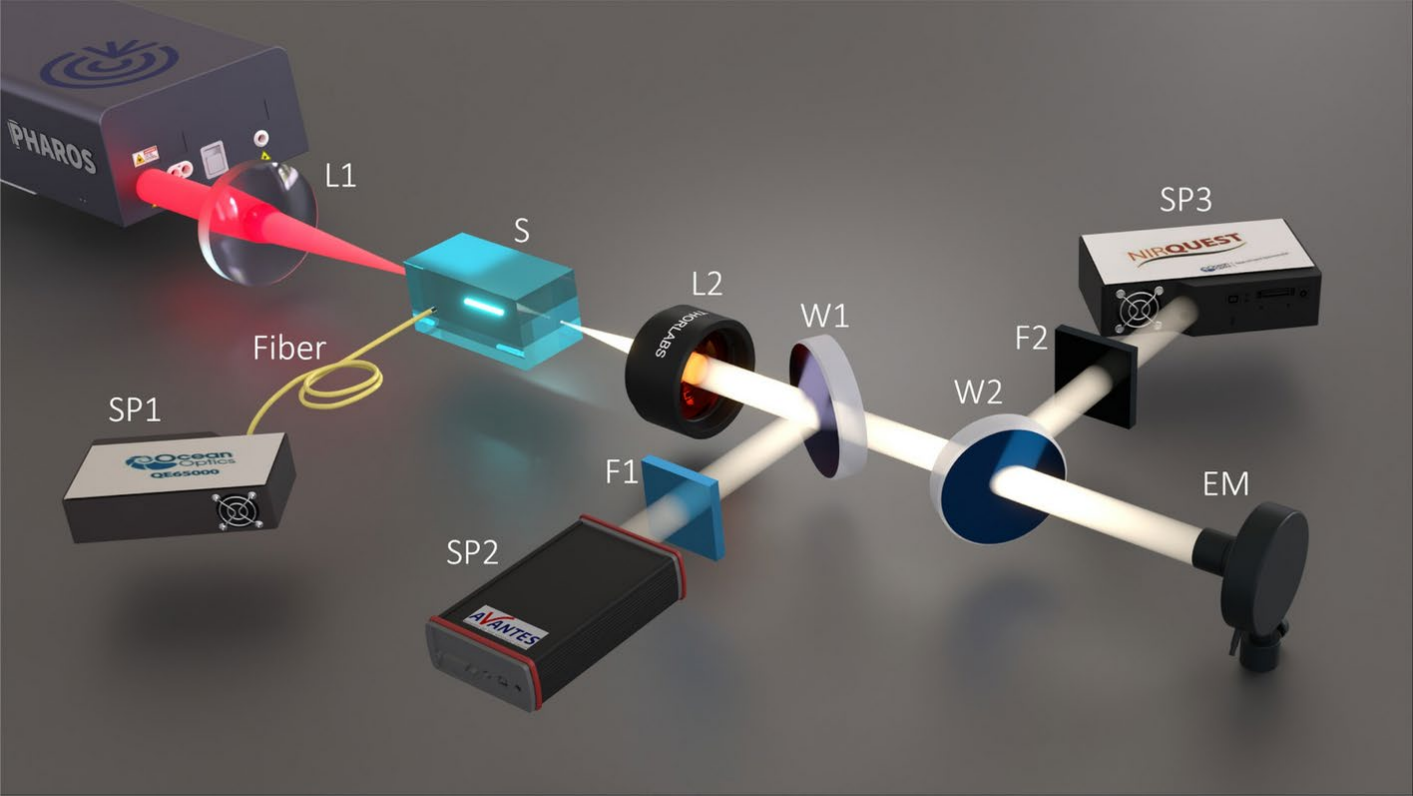
Experimental setup: L1, focusing lens; L2, collimating lens; W1, W2, fused silica wedges; F1, short-pass filter; F2, long-pass filter; SP1, fiber spectrometer, SP2, ultraviolet-near infrared (UV-NIR) spectrometer; SP3, near infrared-shortwave infrared (NIR-SWIR) spectrometer; EM, energy meter.

The simplified experimental setup is depicted in Fig. 1. The laser source we used was an amplified Yb:KGW laser (Pharos, Light Conversion) which provided 180 fs pulses with a central wavelength of 1030 nm at pulse repetition rate which could be tuned up to 200 kHz. The laser beam with a diameter of 5.14 mm (at the 1/$\hbox {e}^{2}$ intensity level) was loosely focused onto the front face of the sample (S) with a fused silica lens L (*f* = +150 mm), which is equivalent to the numerical aperture (NA) of 0.017. The estimated spot size at the beam focus was 41 $\mu$m, with an account of the beam quality factor $\hbox {M}^{2}$ = 1.07, as specified by the laser manufacturer.

The spectral measurements were performed with three spectrometers. Fiber spectrometer SP1 (Ocean Optics QE65000, the detection range 200–900 nm) was used to measure filament-induced crystal luminescence, whose signal was captured from the polished side of the sample. The measurements of SC spectra were performed as follows. The axial portion of the SC radiation was collimated by an achromatic lens L2 (*f* = +75 mm) and, taking the reflections from fused silica wedges W1 and W2, was directed to UV-NIR (AvaSpec-3648, with a detection range of 200–1100 nm, SP2) and NIR-SWIR (NIRQuest-512, with a detection range of 900–2100 nm, SP3) spectrometers to measure the short-wavelength and long-wavelength portions of the SC spectrum, respectively. Both spectrometers were operated simultaneously. In order to increase the dynamic range of spectral measurements, the most intense part of the SC radiation around the pump wavelength was attenuated using appropriate calibrated short-pass (F1) and long-pass (F2) filters placed in front of the UV-NIR and NIR-SWIR spectrometers, respectively. The actual spectra were reconstructed by correcting the measured spectra with an account for the filter transmission functions and for the sensitivity functions of the spectrometers at the data post-processing stage. The spectral dynamics in each sample was measured by fine tuning the pump pulse energy with a computer-controlled attenuator that was integrated in the laser system. Thermal energy meter EM (Ophir 3A-PF-12) was used to measure the SC energy which was thereafter used to calculate the spectral energy density of the SC radiation. All the measurements were performed without any translation of the sample with respect to the pump beam.

## Results and discussion

### Filament-induced luminescence

**Fig. 2:**
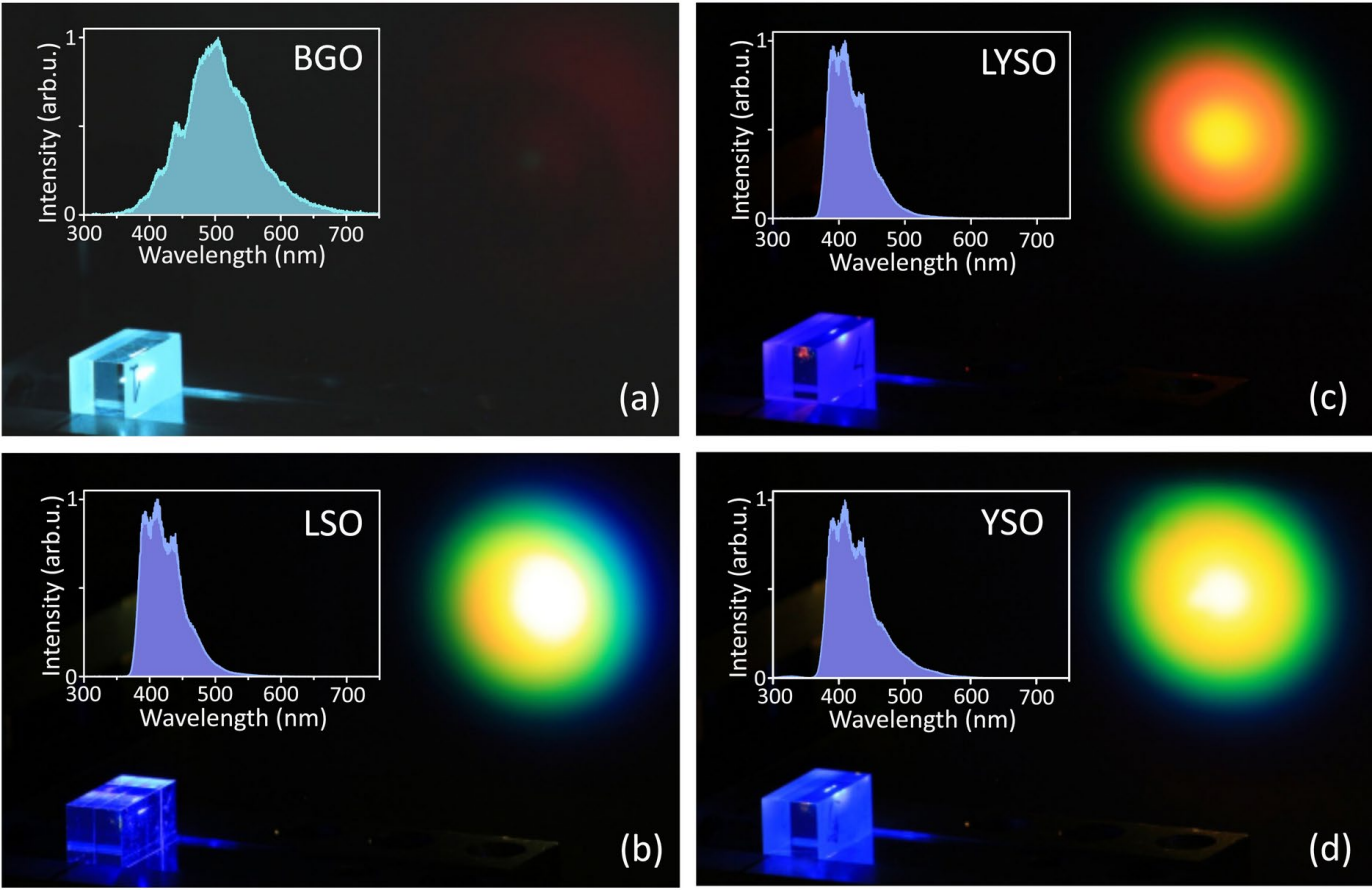
Typical views of supercontinuum generation experiment in (**a**) BGO, (**b**) LSO, (**c**) LYSO, (**d**) YSO crystals. The insets show the respective filament-induced luminescence spectra.

Figure 2 presents the views of BGO, LYSO, LSO and YSO crystals, and their respective SC emission patterns projected onto the paper screen, while the insets show the filament-induced luminescence spectra in these materials, as captured from polished side with a fiber spectrometer, see Fig. 1. All these crystals exhibit remarkably strong filament-induced luminescence, which is easily perceived by the naked eye. In general, in dielectric materials, filament-induced luminescence originates from the relaxation of electrons in the conduction band, which are produced by the multiphoton absorption, and is associated with the relaxation of various transient excited states, such as self-trapped excitons, F centers, impurities, as well as with the emissions due to charge transfer and lattice defects^57^.

BGO crystal produces an exceptionally broadband filament-induced luminescence (Fig. 2a), which spans the entire visible range, with the peak intensity at $\sim 500$ nm, and which is associated with the decay of self-trapped excitons^58^. Filament-induced luminescence spectra in LYSO, LSO and YSO are very similar to each other and cover the 360–550 nm range, with central peaks located at around 410 nm (Fig. 2b–d). GGG also produces strong luminescence in the UV and visible range (not presented in the figure) with the most prominent spectral peaks at 383, 418, 436 and 470 nm. The first two peaks could be associated with luminescence of pure material^59^, while the remaining peaks could be attributed to the luminescence of unidentified dopants, although the supplier specified the sample as undoped. The luminescence of YAG crystal produces strong UV luminescence with a maximum intensity at 300 nm, which is attributed to combined self-trapped exciton and antisite defect-related emissions^60^. Finally, no luminescence in the UV, visible and near-IR was observed in KGW and LT crystals, at least within the detection range of fiber spectrometer (200–900 nm).

Notably, the recorded filament-induced luminescence spectra in the investigated materials are identical to their photoluminescence and radioluminescence spectra, which are produced via direct absorption of high-energy photons in the ultraviolet spectral region and by irradiation of the samples with alpha particles, protons, X-rays and gamma rays, respectively, see e.g.^38,40,61–64^. These are the main approaches to study scintillating properties of the materials, which however have certain limitations for what concerns the penetration depth and risk to modify or damage a relatively large area of the sample. To this end, filament-induced luminescence could be viewed as experimentally simple and cost-effective method for investigating the luminescence properties, such as spectra and decay times (by performing time-resolved measurements) of scintillator crystals. First of all, since the filament-induced luminescence originates from simultaneous absorption of several photons, whose total energy exceeds the bandgap energy of material, it offers easy separation of excitation and emission wavelengths and provides very strong signal despite being emitted over the entire solid angle. The position of the filament track inside the crystal can be easily adjusted in a desired way by varying the pump pulse energy, whereas the intensity clamping in a light filament greatly reduces the risk of optical damage. These features can be helpful for obtaining additional information about crystal homogeneity and reveal the presence and distribution of different dopants in the sample.

### Supercontinuum spectra

**Fig. 3:**
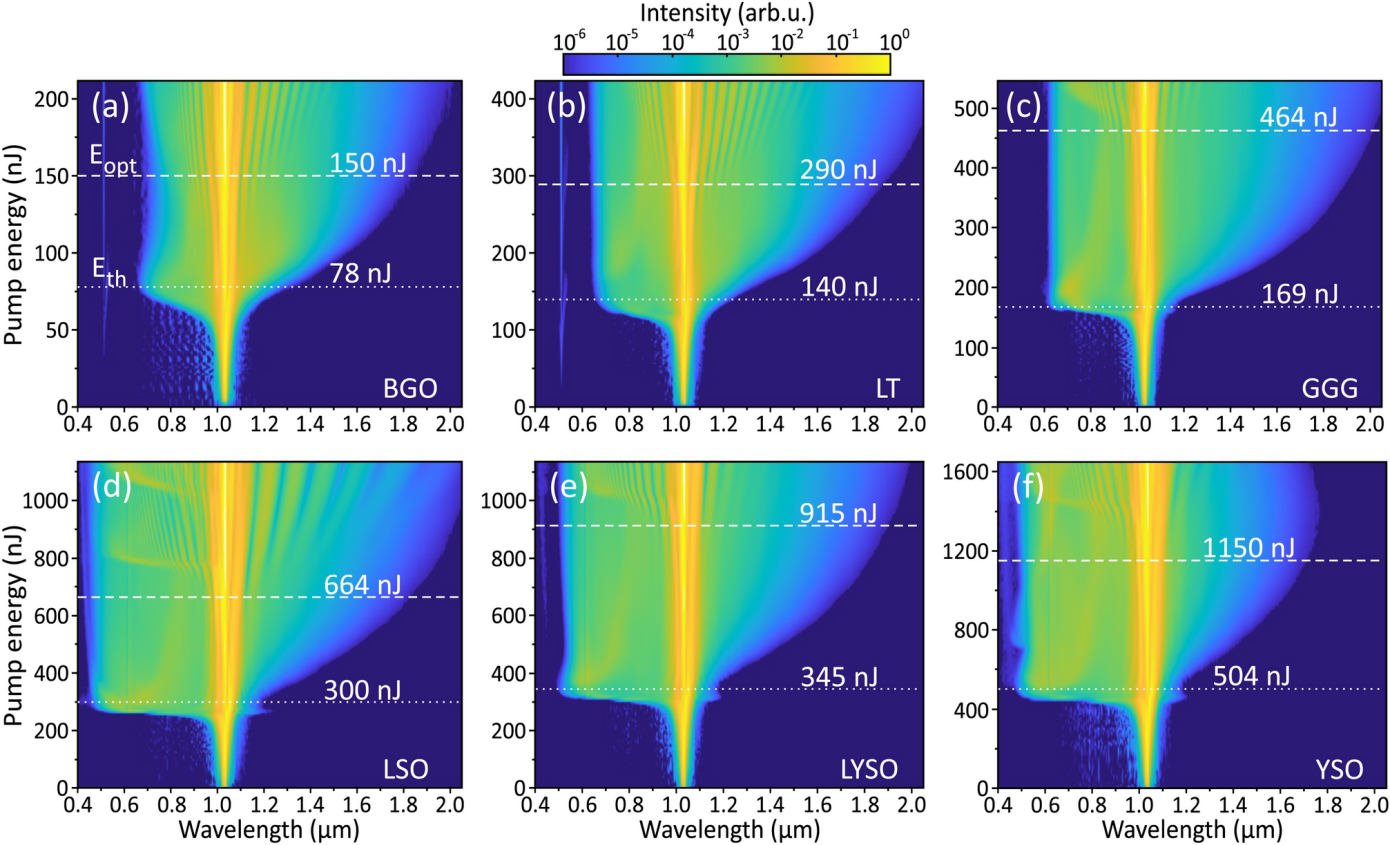
The dynamics of spectral broadening versus the pump pulse energy measured in (**a**) BGO, (**b**) LT, (**c**) GGG, (**d**) LSO, (**e**) LYSO and (**f**) YSO crystals pumped with 180 fs, 1030 nm pulses at 10 kHz repetition rate.

**Fig. 4:**
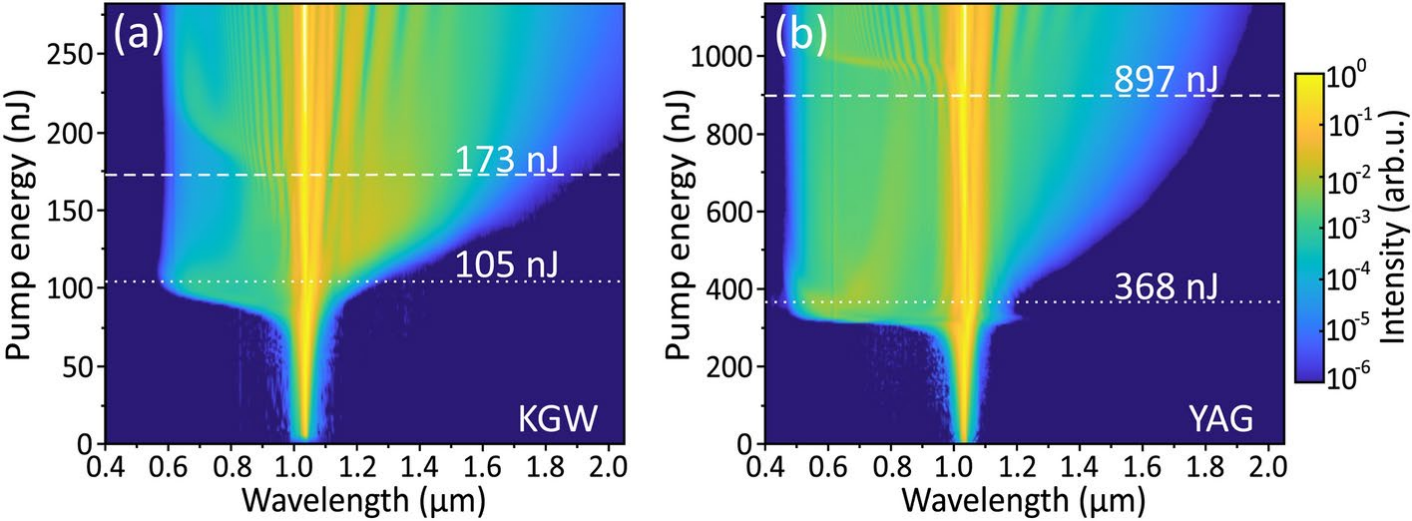
The dynamics of spectral broadening versus the input pulse energy measured in (**a**) KGW, (**b**) YAG crystals.

The screenshots of supercontinua in LYSO, LSO and YSO crystals (Fig. 2b–d) show bright central spots, surrounded by colored rings (conical emission), whose wavelengths decrease from the center to the periphery and reveal broadband nature of SC radiation in these materials. By contrast, no visible light is produced in BGO, where only very faint deep-red conical emission could be barely distinguished (Fig. 2a). Figure 3 presents the detailed dynamics of spectral broadening in BGO, LT, GGG, LSO, LYSO and YSO crystal samples versus the pump pulse energy, where the laser repetition rate was set at 10 kHz. The measured spectral dynamics share relevant common features: an explosive blue-shifted spectral broadening after reaching a certain pump pulse energy level, a much slower red-shifted spectral broadening, which continues more or less throughout the entire investigated energy range, and the occurrence of distinct periodic modulations on both sides of the SC spectrum. The latter feature emerges from filament refocusing, which leads to secondary pulse splitting producing yet another boost of SC generation (which is well distinguishable on the blue-shifted side of SC spectra in Figs. 3c–f, 4a and b), and is a result of interference between the primary and secondary split sub-pulses^65^. This should not be confused with a distinct spectral modulation around the pump wavelength, which is an intrinsic property of SC spectrum associated with interference of partly overlapped spectra of the leading (red-shifted) and trailing (blue-shifted) sub-pulses after primary pulse splitting event at the nonlinear focus, see e.g.^66^ for details.

The threshold energy for SC generation $\hbox {E}_{\textrm{th}}$ was defined as the pump pulse energy, where the blue-shifted spectral broadening quickly settles to a certain cut-off wavelength that remains almost constant even with further increase of the pump pulse energy. The optimum energy $\hbox {E}_\textrm{opt}$, was defined as the pump pulse energy, which produces the largest red-shifted spectral broadening before the onset of periodic modulation in the SC spectrum due to filament refocusing. Both energies are marked by the dashed lines and their values are labeled in the plots. The identical features were also observed in KGW and YAG crystals, whose spectral broadening dynamics are shown for a comparison in Fig. 4.

Note that no trace of filament-induced luminescence in the visible range, that is apparently strong in BGO, GGG, LYSO, LSO and YSO crystals, is detected in their SC spectra, since the luminescence is emitted over the entire solid angle, and a fraction of the luminescence signal which aligns with the beam propagation direction is negligibly small. On the other hand, a weak (at the $10^{-5}$ intensity level), but very clear signal at 515 nm that accompanies SC generation in LT (Fig. 3b) is attributed to phase-mismatched second harmonic generation due to second-order nonlinearity of the crystal. The second harmonic signal is generated also in BGO sample (Fig. 3a), however, this observation is very puzzling, since BGO crystal is optically isotropic due to its cubic lattice symmetry and is not expected to possess any second-order nonlinearity.

It should be noted that the experimentally established SC generation threshold energies and respective peak powers in LSO ($\hbox {E}_{\textrm{th}}$ = 300 nJ, $\hbox {P}_{\textrm{th}}$ = 1.67 MW) and LYSO ($\hbox {E}_{\textrm{th}}$ = 345 nJ, $\hbox {P}_{\textrm{th}}$ = 1.92 MW) were significantly below the calculated value of critical power for self-focusing in these materials ($\hbox {P}_{\textrm{cr}}$ = 2.24 MW that converts to $\hbox {E}_{\textrm{cr}}$ = 403 nJ in both materials, see Table 1). This result implies that two parabolic band model provides underestimated $\hbox {n}_{\textrm{2}}$ values of LSO and LYSO. To solve this issue, we evaluated the $\hbox {n}_{\textrm{2}}$ values of LSO and LYSO by comparing the experimentally established threshold energies for SC generation in these materials with that of YAG, bearing in mind an identical beam focusing condition, equal sample lengths, similar bandgaps and an apparent similarity of SC generation performances in LSO (Fig. 3d), LYSO (Fig. 3e) and YAG (Fig. 4b). More specifically, this was done by taking the ratio between the corrected (after subtracting Fresnel reflection from an uncoated input face) threshold energies for SC generation measured in LSO and LYSO (labelled in the respective plots) and so the corrected threshold energy in YAG, whose $\hbox {n}_{\textrm{2}}$ value is reliably established experimentally, see Table 1. Such crude estimation gives the nonlinear refractive index values of $7.6\times 10^{-16}$ $\hbox {cm}^2$/W and $6.6\times 10^{-16}$ $\hbox {cm}^2$/W of LSO and LYSO, respectively. These new $\hbox {n}_{\textrm{2}}$ values yield the respective critical powers for self-focusing of 1.18 MW and 1.36 MW in LSO and LYSO, which appear very reasonable estimates and are added in Table 1.

**Fig. 5:**
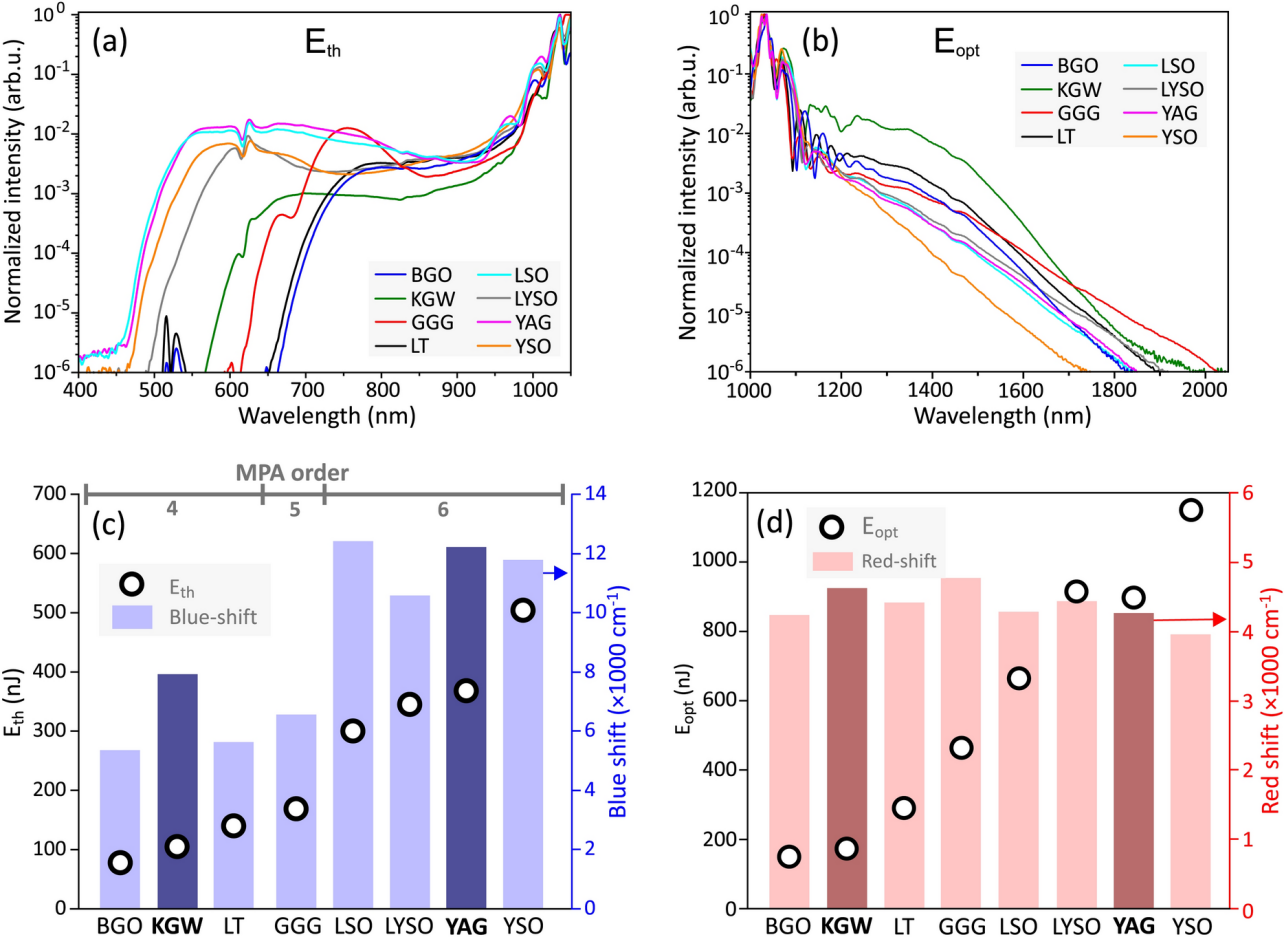
(**a**) The short-wavelength parts of SC spectra measured at threshold energies $E_{\textrm{th}}$ of SC generation. (**b**) The long-wavelength parts of SC spectra recorded at optimal pump pulse energies $E_{\textrm{opt}}$. (**c**) Threshold energies and corresponding spectral blue-shifts. (**d**) Optimal pump pulse energies and corresponding spectral red-shifts. Darker shadings in (**c**) and (**d**) highlight the results of KGW and YAG, which served as reference nonlinear materials.

The results of SC generation in all the tested materials are summarized in Fig. 5. Figure 5a compares the blue-shifted portions of SC spectra in the visible and near IR range, recorded at threshold energies of SC generation. With 1030 nm pumping, YAG, LSO, LYSO and YSO produce the largest spectral blue shifts, with the blue cut-off wavelengths (estimated at the $10^{-6}$ intensity level) below 500 nm: LSO at 450 nm, YAG at 460 nm, LYSO at 490 and YSO at 465 nm. Modest spectral blue-shifts were measured in KGW (567 nm) and GGG (610 nm), while LT and BGO did not produce any SC in the visible range, with the cut-off wavelengths at 650 nm and 664 nm, respectively. Note also the spectral peaks around 515 nm produced in the latter two crystals, which are attributed to the second harmonic. Figure 5b compares the red-shifted portions of SC spectra produced with optimal pump pulse energies. All the tested materials, except YSO, demonstrated reasonably large spectral red-shifts, which exceeded 1800 nm. In that regard, GGG, KGW and LYSO produced the largest spectral red-shifts extending up to 2025 nm, 1965 nm and 1910 nm, respectively (estimated at the $10^{-6}$ intensity level), exceeding the red-shift produced in YAG (1850 nm). In Fig. 5c the spectral blue shifts are presented in the order of increasing threshold energies, which align well with the calculated values of critical power for self-focusing (see Table 1). The top axis of the plot indicates the order of multiphoton absorption *K*, which was evaluated using an expression $K=\langle U_g/\hbar \omega _0 \rangle +1$, where $\hbar \omega _0 = 1.2$ eV is the photon energy at the laser carrier frequency and brackets denote the integer part of the ratio. More specifically, the pump pulse experiences four-photon absorption in BGO, LT and KGW, five-photon absorption in GGG, and six-photon absorption in LSO, LYSO, YSO and YAG. The spectral data are in line with the universal trend of the SC blue-shift dependence on the material bandgap, and so on the order of MPA, which in turn defines the clamping intensity of the filament^6,7^. Only KGW crystal drops off this general trend, demonstrating that this nonlinear material despite its relatively narrow energy bandgap produces unproportionately large blue-shifted spectral broadening. The general increase of optimum pump pulse energy for the optimized red-shifted spectral broadening follows very similar trend as the threshold energy, however, there is no bandgap dependence of the spectral red-shift, as demonstrated in Fig. 5d. The narrow bandgap nonlinear materials: BGO, LT, KGW and GGG produce reasonably large red-shifted spectral broadenings which are comparable or even larger than those measured in LSO, LYSO and YAG.

**Fig. 6:**
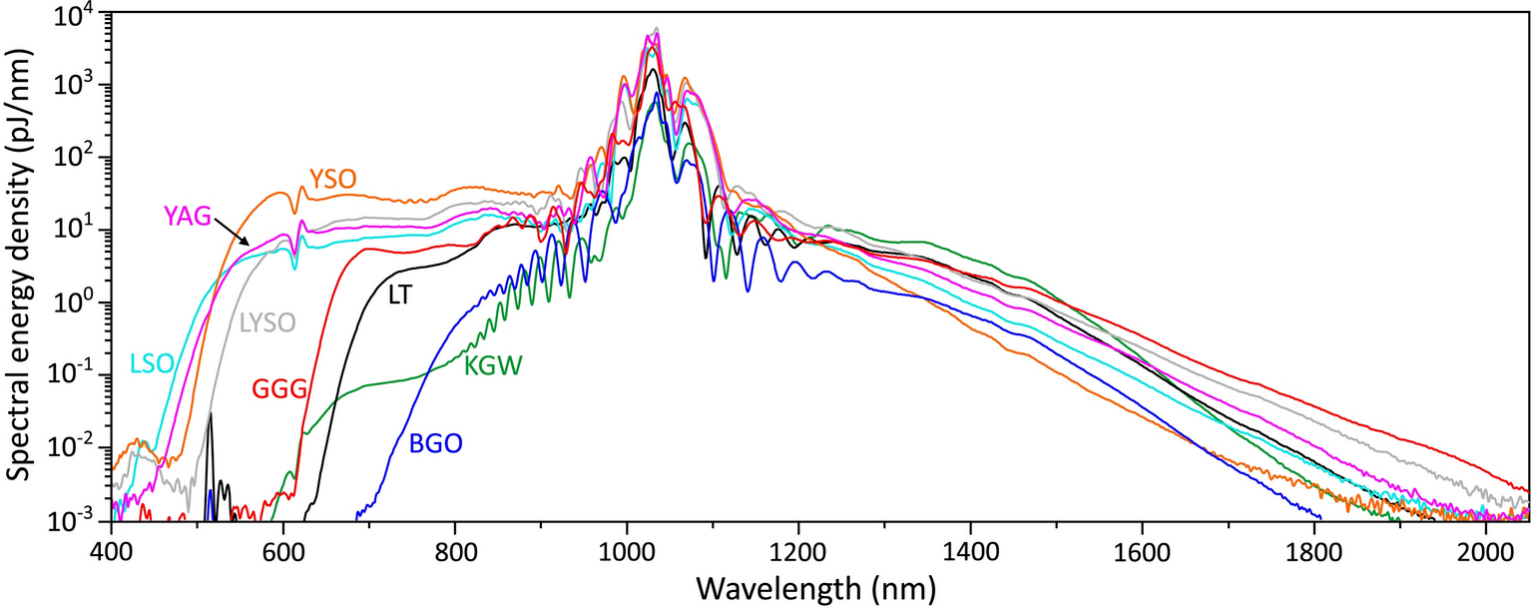
Comparison of spectral energy densities of supercontinua produced in the conditions of optimal pumping in the investigated materials.

The red-shifted part of the SC is of practical importance, especially for what concerns the performance of broadly tunable optical parametric amplifiers pumped by the fundamental harmonics of Yb lasers. The lack of seed photons in the long-wavelength region of the SC spectrum limits the amplification efficiency and results in pulse-to-pulse energy fluctuations around the degeneracy region of the optical parametric amplifier. This aspect is quantified in Fig. 6a by plotting the SC spectra in terms of spectral energy density, which was estimated by measuring the energy of the axial part of SC radiation (which is the only practically useful part of SC emission). KGW crystal produces the highest spectral energy density in the 1200–1450 nm range, as noted in a recent study^30^. The red-shifted part of the SC spectrum in BGO has the lowest spectral energy density, which is 1–2 orders of magnitude lower than in the rest of the materials. However, very low energy threshold of SC generation, as well as low optimal pump energy in BGO, should be pointed out. The estimated spectral energy densities produced in LSO and LYSO are quite similar to YAG throughout the major part of the SC spectrum. The most interesting finding in that regard is GGG crystal, which, despite very moderate blue-shifted spectral broadening, produces the largest spectral red-shift and the highest spectral energy density in the 1600–2000 nm range, which is almost an order of magnitude higher than that produced in YAG.

### Performances at high laser repetition rates

**Fig. 7:**
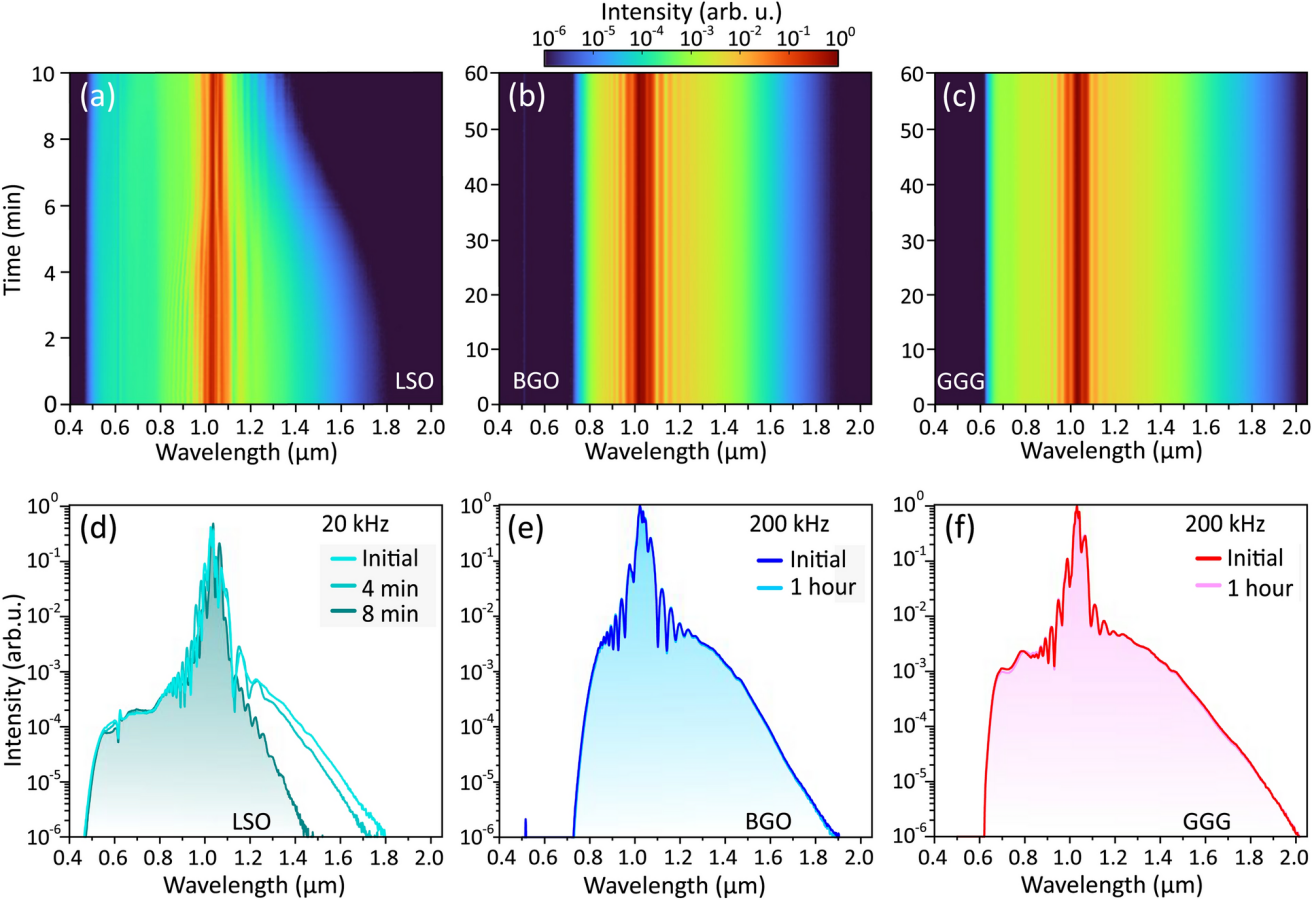
Long-term dynamics of the SC spectra generated at optimum pumping conditions in (**a**, **d**) LSO, (**b**, **e**) BGO and (**c**, **f**) GGG. The pulse repetition rate were 20 kHz in LSO, and 200 kHz in BGO and GGG.

Finally, we investigated the long-term performances of SC generation in crystal samples under optimal pumping conditions at higher laser repetition rates. In doing so, the crystal samples were not translated and kept in a stationary position with respect to the pump beam during the entire measurement. The examples illustrating robust long-term performances of SC generation in KGW and YAG crystals at high laser repetition rates could be found elsewhere. More specifically, stable performance of KGW crystal at 2 MHz pulse repetition rate under very similar pump beam focusing geometry was reported recently^30^, whereas YAG crystal demonstrated stable performance for several hours at 200 kHz pulse repetition rate even with very tight focusing of the pump beam (NA=0.085)^32^. The long-term performance tests in LSO, LYSO and YSO revealed gradual narrowing of the SC spectra on the long-wavelength side that occurred after a certain exposure time and could be attributed to the optical degradation of these materials. For instance, in LSO, the onset of spectral narrowing was observed after $\sim 20$ minutes at a laser repetition rate of 10 kHz, while at a laser repetition rate of 20 kHz, stable SC generation performance was observed just for approximately 2 min. An example of such behaviour is shown in Fig. 7a. The character of spectral changes is highlighted in Fig. 7d, which shows how the SC spectrum on the long-wavelength side shrinks considerably, from 1800 nm to 1450 nm, just after 8 minutes of exposure time in a stationary sample. LT crystal demonstrated somewhat better long-term performance: at pulse repetition rates of up to 100 kHz no narrowing on either side of the SC spectrum was detected. However, rapid narrowing of the SC spectrum in LT crystal was observed after the first 5 minutes of operation when the pulse repetition rate was set at 200 kHz (data not shown). In contrast to the above materials, BGO and GGG demonstrated excellent SC generation performances without any spectral changes at a pulse repetition rate of 200 kHz, as attested by the time series of SC spectra in BGO and GGG, shown in Fig. 7b and c, respectively. Figure 7e and f outline identical shapes of SC spectra at the beginning and at the end of 1 hour-lasting measurement in both materials.

## Conclusions

In conclusion, we demonstrated that undoped scintillator crystals: BGO, GGG, YSO, LSO and LYSO are efficient nonlinear materials for broadband SC generation in the near-infrared and short-wave infrared spectral ranges, when pumped by 180 fs fundamental harmonic pulses from an amplified Yb:KGW laser. YSO, LSO and LYSO produced two octave-spanning SC spectra, which compare to SC spectra produced in YAG. These materials exhibit quite similar performances to YAG also in terms of threshold and optimal pump pulse energies for SC generation. However, for what concerns the possibility to operate at high laser pulse repetition rate, YSO, LSO and LYSO show durable operation only at 10 kHz and quickly degrade (within a few minutes) at 20 kHz. Besides the relatively low SC generation threshold (169 nJ), GGG demonstrated superb performance characteristics in the short-wave infrared with the optimal pump pulse energy of 464 nJ, which is two times lower than in YAG: the largest red-shifted spectral broadening and the highest spectral energy density, outperforming KGW and YAG crystals in that aspect. Although BGO and LT produced the narrowest SC spectra (slightly less than 1.5 optical octaves), they could be viewed as very attractive nonlinear materials for broadband SC generation with pumping at longer laser wavelengths. Owing to its large nonlinearity, BGO exhibited the lowest SC generation threshold (78 nJ) among the investigated materials, which is 25 % lower than in KGW and could be considered as excellent nonlinear material for very low-threshold SC generation. The observed stable and durable damage-free performance of BGO and GGG at 200 kHz pulse repetition rate suggests that these nonlinear materials have a potential for SC generation at even higher, MHz, repetition rates. Moreover, these materials provide SC radiation with an excellent set of parameters that are essential for applications in high speed spectroscopy and imaging that employ bulk-generated SC, see e.g.^17–20^.

The spectral measurements of filament-induced luminescence suggest that scintillating properties of materials could be readily studied using femtosecond near-infrared light source via multiphoton excitation in the filamentation regime, which allows easy separation of excitation and emission wavelengths and low risk of optical damage.

## Data Availability

The datasets used and/or analysed during the current study available from the corresponding author on reasonable request.
